# Insulator Leakage Current Prediction Using Hybrid of Particle Swarm Optimization and Gene Algorithm-Based Neural Network and Surface Spark Discharge Data

**DOI:** 10.1155/2022/6379141

**Published:** 2022-08-25

**Authors:** Phuong Nguyen Thanh, Ming-Yuan Cho

**Affiliations:** ^1^Department of Electronic Engineering, National Kaohsiung University of Science and Technology, Kaohsiung, Taiwan; ^2^Department of Electronic and Electrical Engineering, Nha Trang University, Nha Trang, Khanh Hoa, Vietnam

## Abstract

This study proposes a new superior hybrid algorithm, which is the particle swarm optimization (PSO) and gene algorithm (GA)-based neural network to predict the leakage current of insulators. The developed algorithm was utilized for the online monitoring systems, which were completely installed on the 69 kV and 161 kV transmission towers in Taiwan. This hybrid algorithm utilizes the local meteorological data as input parameters combined with the extracted enhanced data: the percentage of spark discharge areas and the brightness change in the image of the discharge phenomenon. These data with a high correlation with the leakage current are utilized as input vectors to improve the accuracy and effectiveness of the developed hybrid model. The performance of the developed algorithm is compared with a traditional PSO-based neural network and backpropagation neural network (BPNN) to evaluate and analyze. The comparative simulation results prove the effectiveness of the combination of hybrid PSO-GA-based neural network and surface discharge data, which achieved a maximum improvement of 38.54% MSE, 10.62% MAPE, and 3.41% *R* square for 161 kV data and 39.28% MSE, 12.62% MAPE, and 1.61% *R* square for 69 kV data. Moreover, the data with enhanced inputs outperform the traditional data in most benchmark factors, improving the accuracy and effectiveness in defining the deteriorative insulators. The developed methodology with a noticeable improvement was utilized in the online monitoring system to reduce the operational and maintenance cost of transmission lines in Taiwan Power Company.

## 1. Introduction

The power transmission insulators are often polluted on insulations' surfaces, commonly affected by salt fog, causing severe accidents to the transmission system. The polluted insulators are typically deteriorated by flashover on the surface, which causes a severe power outage and group tripping in the power system. The insulator's condition could be classified by measuring the leakage current on the surface [1–3]. Many studies have been developed to predict the insulator leakage current by employing neural networks and meteorological data [4–13]. Zhicheng et al. have proved the strong correlation between leakage current and other meteorological data, such as humidity and rainfall. Gao et al. applied the backpropagation neural network to predict the leakage current, using temperature, humidity, and rain as input parameters. In previous studies, the author has indicated the effectiveness of combining PSO-based neural networks and surface spark data, increasing accuracy in forecasting the leakage current [14]. The PSO-based neural network has improved accuracy and effectiveness compared with the other persistent models, such as the support vector machine, the radial basis function neural network (RBFN) with *K*-means cluster, and the backpropagation neural network in forecasting the leakage current [14]. Besides, the simulation results have demonstrated that the surface spark data, which are the percentage value of SSDD and the change of spark brightness, could improve the effectiveness and accuracy in predicting the leakage current [14]. Ozgur et al. utilized the PSO-based neural network to predict the short-term load power, which achieved a faster convergence in both MAE and MSE with the database of North Cyprus [15]. Wen proposed the hybrid PSO and the combined BPNN and RBF for predicting short-term wind power [16]. The proposed method was experimentalized on the Taichung coast data in Taiwan, which proved the more accurate and reliable methodology compared with RBFNN and persistence BPNN. Xie et al. developed the combined algorithm between the Elman NN and PSO for predicting the short-term power, illustrating the PSO-ENN's effectiveness compared with other traditional networks [17]. Therefore, the combination of PSO and the neural network proves the higher performance and better accuracy than traditional neural networks.

The gene algorithm (GA) considerably outperformed the existing adaptive algorithm and proved the advantage of running efficiently to solve several problems in different fields [18]. The GA could solve more complicated technological and scientific difficulties with the natural evolutionary process. However, the GA is normally utilized with another local search algorithm because of its difficulties in effectively exploring the solution space [19]. Therefore, combining a global search optimization algorithm and GA search methodology could improve the effectiveness and enhance the performance of both searching methodologies. Many previous studies utilized the global search methodology, PSO, and local search algorithm, GA, which proved a fast local search strategy [20–25]. Sanjib et al. proposed neural networks trained by the PSO and GA, which improved convergence time [26]. Moradi et al. utilized the combined GA and PSO for optimal location and sizing of distributed generation sources in distribution systems [27]. The proposed methodology has minimized the losses, increased the voltage stability, and improved the voltage regulation index for 33 and 69 bus systems [27]. Shiwei et al. presented the hybrid PSO-GA optimized radial basis function neural network to predict annual electricity demand [28]. Keo and Zahara proved that the hybrid GA and PSO achieved superiority compared with other solutions in terms of convergence rates and the optimal global results in the experiments with 17 multimodal functions [29]. The combination of GA and PSO has synthesized the crossover and mutation processes in the GA and the particle evolution of the PSO in handling different kinds of problems [29]. Valdez et al. combined the hybrid PSO-GA and fuzzy logic, which outperformed the GA and PSO separately with 10 mathematical functions for parameter tuning [30]. Pedram and Jon have integrated the GA and PSO for feature selection methodology, which could provide the most informative features in the Indian Pines hyperspectral data [31]. The hybrid methods obtained better approaches compared with other algorithms within limited CPU processing time. Ahmed and Mohamed have developed the hybrid PSO and GA for solving the energy function of the molecule, which is 1000 dimensions [32]. The proposed hybrid method was compared with the other nine benchmark algorithms in minimizing the loss function, which proved the promising and efficient method in converging to the global minimum and faster velocity to the near-global solution. Wu et al. have developed the hybrid PSO-GA for selecting the parameters for the RBF neural network in predicting rainfall [33]. The comparison of HPSO-GA and pure GA proved that the hybrid method obtained a more effective exploration ability by avoiding premature convergence and achieving higher forecasting accuracy. Therefore, the combination of PSO and GA leads to an efficient algorithm to solve more difficult optimized problems. However, hybrid PSO-GA has no application in predicting the insulator's leakage current in the online monitoring system.

In this paper, the predicting model in the online monitoring system could be enhanced by using a hybrid of PSO- and GA-based neural networks, which utilized the surface spark data as an enhancement input parameter. The hybrid PSO-GA performance is compared with the previous model, which is the PSO-based neural network and backpropagation neural network (BPNN). These models used the input data, which are the meteorological and surface spark data, to evaluate the effectiveness and accuracy of the proposed algorithm. The significant contribution of this paper is to evaluate the performance of hybrid PSO- and GA-based neural networks compared with the PSO-based neural network model and backpropagation neural networks in predicting the leakage current of 69 kV and 161 kV insulators. In addition, the methodology utilized the enhanced input data for these predicting models, which are the meteorological and surface spark discharged data. The data with enhanced inputs outperform the traditional data, which only utilized the weather factors. The paper has strongly proved the improving effectiveness of predicting performance by combining the PSO-GA-based neural network and surface spark data. In addition, the correlation matrix between leakage current and weather parameters is presented in this research. In the next section, the methodology presents the extraction of surface spark data and the detailed algorithms of a hybrid PSO-GA-based neural network. The third part introduces the experiment results of these algorithms with different error metrics. This part also evaluates the performance of the hybrid PSO-GA-based neural network with other traditional models.

## 2. Methodologies

### 2.1. The Online Monitoring System in Predicting the Leakage Current

**Figure 1:**
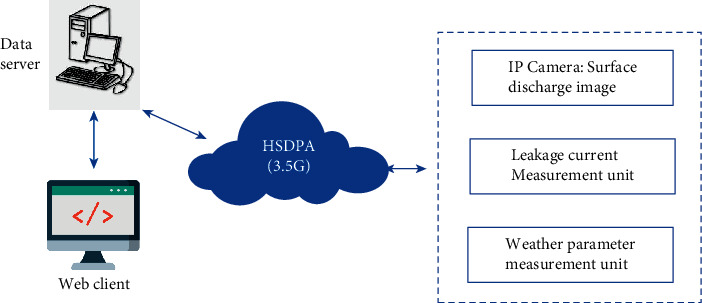
The general structure of online monitoring system.

The online monitoring system has been established for the 69 kV and 161 kV transmission towers in Yunlin County of Jianan District, which are seriously affected by salt fog pollution. The system consists of a front-end web client, the data server, the 3.5G communication system, the surface discharge image collecting system, the leakage current measurement unit, and the weather parameter measurement units. The general structure of this system is described in Figure 1. The system uses the IP camera to collect the surface discharge image in real time. Besides, the weather parameter measure unit will collect some essential weather parameters, which significantly affect the leakage current: the temperature, the humidity, the dew point, the wind speed, wind direction, and the rainfall. These weather parameters are utilized to predict the leakage current in the artificial neural network model. The leakage current measurement unit will automatically collect the surging value of leakage current for the testing and validation of the predicting model. In addition, the value of leakage current is utilized for training the neural network model. The IP camera module will be installed in the 69 kV and 161 kV transmission towers to collect the image of the surface discharge of insulators. The collecting weather parameters and images are transferred to the data server through the 3.5G communication network. The neural network model is integrated into the data server to predict the leakage current. The user could easily observe the value of leakage current and other weather parameters through a front-end web application.

**Figure 2:**
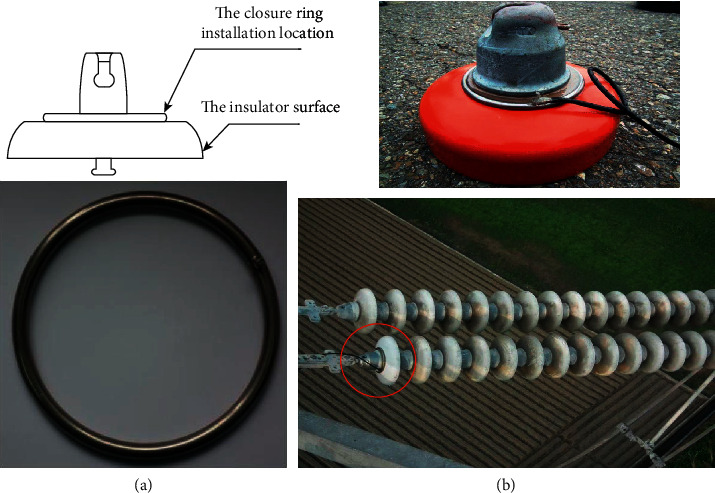
The installation of leakage current measuring rings on insulators. (a) The actual leakage current ring. (b) Installation leakage current measuring rings on insulators.

**Figure 3:**
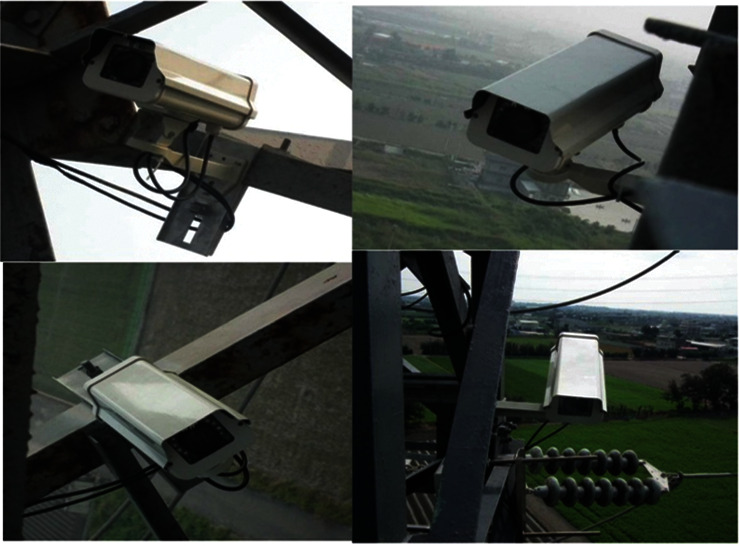
The installation of IP cameras on transmission tower.

In this monitoring system, the weather parameters, which are temperature, humidity, dew point, wind speed, air pressure, and rainfall, are collected every hour. The weather data are divided into the training and testing dataset.  Table 1 summarizes the maximum, the minimum, the average, and standard deviation of these weather parameters which have been collected. The leakage currents are measured and collected for training and testing purposes by utilizing the leakage current cut-off ring. The installation of seamless stainless steel on insulators is shown in Figure 2. This ring was installed on the insulator surface sheet, which is the closest to the transmission tower. The ring was installed firstly on the insulator by AB glue, and then it can be installed on the transmission line.  Figure 3 shows the complete picture of the on-site installation of the IP camera. The camera is fixed on the transmission tower structure by utilizing the L-shaped clips. The design structure allows construction personnel or maintenance to quickly install the IP camera without damaging the structure of transmission towers.

### 2.2. Enhanced Input: Surface Spark Discharged Data

**Figure 4:**
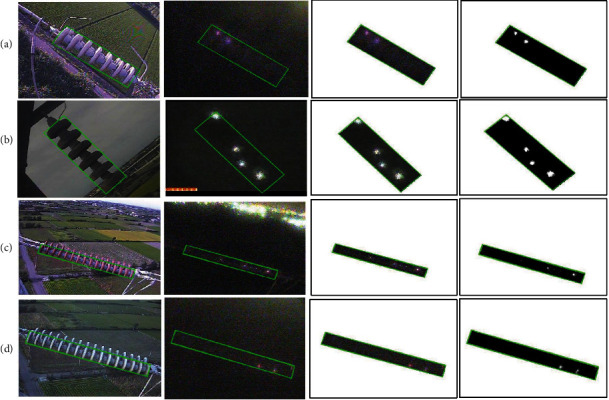
From left to right: the process of calculating the surface spark data: (a) and (b) for 69 kV insulators, (c) and (d) for 161 kV insulators.

The data acquisition station could automatically collect the meteorological data within a specified period [14]. The meteorological data were utilized as an input parameter to forecast the leakage current, such as the temperature, the wind speed, the dew point, rainfall, and humidity. Besides the meteorological data, the online monitoring system also collects the surface spark discharge phenomenon images. These capture images will be processed to extract additional input parameters for predicting leakage current, as shown in Figure 4. If a flashover phenomenon occurs at night, this discharge phenomenon could be observed on the surface of the insulator. The region of interest (ROI) of the discharged phenomenon is identified in the image and is utilized to extract the surface spark data: the percentage of spark area in equation (1) and the change in the brightness of ROI in equation (2) [14]. The processes of extracting additional input parameters are clearly illustrated in Figure 4. When the surface spark discharge images are captured, these captured images are transmitted to the server by a 3G network. The server system will define the ROI and eliminate other regions on the image. The spark area will be calculated by counting the number of white pixels in the ROI, which represents the spark discharge area. Besides, the brightness change is calculated based on the luminance component value in the ROI before and after the spark discharge phenomenon. The average luminance component is calculated to extract the brightness change in ROI. These additional extracted parameters are applied to predict leakage current in the online monitoring system. The input layer of neural networks consists of nine parameters, which has seven weather parameters: the temperature, the humidity, the dewpoint, the wind speed, the wind direction, air pressure, and the rainfall, and two enhanced parameters: the percentage of SSDP and the change of brightness SSDP. Besides, the data also are divided into two models: data model 1, which only comprises the weather parameters, and data model 2, which comprises the weather parameters and the enhanced parameters. These data models will be used to compare the accuracy and effectiveness in predicting leakage current with different artificial neural network models.

(1) $$\begin{array}{c} {\text{Percentage}}_{\text{SSDP}}=\frac{\text{Area of Spark}}{\text{Area of ROI}}, \end{array}$$

(2) $$\begin{array}{c} {\text{Brightness}}_{\text{SSDP}}=\frac{\left|{\text{Average}}_{I\_\text{new}}-{\text{Average}}_{I\_\text{old}}\right|}{{\text{Average}}_{I\_\mathrm{old}}}. \end{array}$$

The leakage current is strongly dependent on the collected weather parameters, which is necessary to identify in the prediction field. The associated meteorological variables and extracted enhanced inputs provide accurate projections in predicting leakage current. The Pearson correlations between input parameters and the leakage current are presented in Table 2. The Pearson correlations are utilized to determine which input parameters have significant relevance on each other's and remove irrelevant data [34, 35]. This correlative coefficient measures the linear dependence between two random vectors. This coefficient is widely utilized to reflect the linear correlation between two normal continuous data, as in the following equation:

(3) $$\begin{array}{c} r_{xy}=\frac{\sum \left(x_{i}-\bar{x}\right)\sum \left(y_{i}-\bar{y}\right)}{\sqrt{{\sum \left(x_{i}-\bar{x}\right)}^{2}}\sqrt{{\sum \left(y_{i}-\bar{y}\right)}^{2}}}. \end{array}$$

The Pearson correlation ranges between −1 and 1, and its absolute value reflects the linear regression between two random variables [36, 37]. It could be observed from Table 2 that:

- The extracted percent SSDP and brightness SSDP have positively correlate with the leakage current. The higher leakage current is associated with greater enhanced parameters values, which is confirmed by 0.22 and 0.67 Pearson correlations. The brightness SSDP has a more significant impact on the leakage current than the percent SSDP, proved by the Pearson correlation values.
- The temperature, dew point, and wind direction negatively correlate with the leakage current. The higher these parameters are linked to a smaller leakage current by the observed Pearson correlations, which are −0.15, −0.44, and −0.17, respectively.
- The humidity, wind speed, air pressure, and rainfall have a positive linear correlation with leakage current, providing relevant information in the predicting model. These parameters were proved as the most relevant input parameters with leakage current in previous studies [37, 38]. These weather parameters could provide more consistent characteristics and are considered potential system factors for predicting the leakage current.

Therefore, based on the most relevant Pearson correlations between collected parameters, the leakage current of the insulator could be predicted as a function of percent SSDP, brightness SSDP, temperature, humidity, dew point, wind direction, wind speed, air pressure, and rainfall. These vital correlation parameters could provide the most appropriate information and improve the accuracy and performance of predicting model.

### 2.3. Gene Algorithm

The genetic algorithm (GA) is a heuristic stochastic search algorithm and a powerful tool of optimization, which has good global searching ability based on crossover and mutation of evolutional biological principles [39–41]. The GA could achieve the best fitness solution without the gradient information of error functions. The GA has multiple points' searching capacity, which discriminates from other searching methodology [42]. The initial population of GA is randomly generated. Each population is the candidate solution for the problem, which is called a chromosome and contains a vector of genes. The fitness value, which is based on requirements for each problem, is used to evaluate each individual's fitness. The goal of the evolutional genetic operation, which is crossover and mutation, is to search for an optimal solution to the complex problem. Individuals exchanged their partial gene fragments to obtain the new generation in the crossover process. This evolutional process replaces specific codes in each gene and creates new populations with a better fitness function value. The selection of individuals as “parents,” which depended on the value of their fitness function, plays a significant role in GA. In this paper, the roulette wheel selection is utilized between several selection schemes [43, 44]. The two individuals are selected and exchange their partial gene fragment between crossover points. The new populations could inherit superior genetic information with better optimum solutions than old generations [43, 44]. In this paper, arithmetic is utilized between different crossover processes, including simple crossover, heuristic crossover, and arithmetic crossover. Let us assume two parent chromosomes ${\overset{⟶}{X}}^{1}=\left(x_{1}^{1},\ldots ,x_{i}^{1},\ldots ,x_{n}^{1}\right)$ and ${\overset{⟶}{X}}^{2}=\left(x_{1}^{2},\ldots ,x_{i}^{2},\ldots ,x_{n}^{2}\right)$ are selected for the arithmetic crossover process; the two new generations ${\overset{⟶}{Y}}^{1}=\left(y_{1}^{1},\ldots ,y_{i}^{1},\ldots ,y_{n}^{1}\right)$ and ${\overset{⟶}{Y}}^{2}=\left(y_{1}^{2},\ldots ,y_{i}^{2},\ldots ,y_{n}^{2}\right)$ are built as following equation, where *r* is a random number in the interval [0, 1].

(4) $$\begin{array}{c} {\overset{⟶}{Y}}^{1}=r{\overset{⟶}{X}}^{1}+\left(1-r\right){\overset{⟶}{X}}^{2},\\ {\overset{⟶}{Y}}^{2}=r{\overset{⟶}{X}}^{2}+\left(1-r\right){\overset{⟶}{X}}^{1}. \end{array}$$

In the mutation operation, the gene randomly alters its chromosome element according to the mutation probability, *P*_*m*_ [45]. The nonuniform mutation is described in equation (5). In this equation, *a*_*i*_ and *b*_*i*_ are the mutation boundary, *G* and *G*_max_ are the numbers of current and maximum generation, *r*_1_ and *r*_2_ are random numbers, and *b* is the degree of dependency of iteration [44].

(5) $$\begin{array}{c} x_{i}^{\prime }=\left\{\begin{array}{cc} x_{i}+\left(b_{i}-x_{i}\right)f\left(G\right), & \text{if }r_{1}<0.5,\\ x_{i}-\left(x_{i}+a_{i}\right)f\left(G\right), & \text{if }r_{1}\geq 0.5,\\ x_{i}, & \text{if }x_{i}\notin \;\left[a_{i},b_{i}\right], \end{array}\right.\\ f\left(G\right)={\left(r_{2}\left(1-\frac{G}{G_{\max}}\right)\right)}^{b}. \end{array}$$

The probabilistic nature of crossover and mutation reproduces the most substantial individual in the population, which has the more excellent fitness function. The best-fit string of genes is derivable to transfer to the next generation. The performance of GA is susceptible to the initial individuals of the population, which are typically randomly selected. Therefore, the GA is often modified to combine with another algorithm to improve the effectiveness of practical problems. The GA could be developed and simulated by using MATLAB.

### 2.4. Particle Swarm Optimization Algorithm

The PSO-based neural network is deployed to forecast the leakage current in the online monitoring system. The PSO algorithm is a robust optimization algorithm that generally outperforms, efficiently searches for better solutions, and has faster convergence compared with other optimization algorithms [25, 46, 47]. The PSO iteratively accelerates particles towards the personal and global best, which have been utilized in many applications [48, 49]. The particle position and velocity are iteratively updated by using equations (6) and (7), respectively. The updating velocity in equation (6) includes three main elements: the momentum component, the personal sector, and the global component. The number of PSO individuals typically influences the searching capability for the global optimum solution. The more significant density of the initial population increases the collaborative searching capability and improves the convergent speed of particles.

(6) $$\begin{array}{c} v^{\left(i,k+1\right)}=w*v^{\left(i,k\right)}+C_{1}*\text{rand}\left(1\right)*\left(x_{p}^{\left(i,k\right)}-x^{\left(i,k\right)}\right)+C_{2}*\text{rand}\left(1\right)*\left(x_{G}^{\left(k\right)}-x^{\left(i,k\right)}\right), \end{array}$$

(7) $$\begin{array}{c} x^{\left(i,k+1\right)}=x^{\left(i,k\right)}+v^{\left(i,k+1\right)}. \end{array}$$

#### 2.4.1. The Back Propagation Neural Network

The backpropagation neural network (BPNN) structure has three main layers: an input layer, a hidden layer, and an output layer connected through neurons and weights. In addition, the weight values, *w*_*ji*_, and bias values, *b*_*ij*_, between neurons are randomly generated as initiative values. The training process has two main steps: the feedforward and backpropagation processes. In the feedforward steps, the output vector, *Y*_*i*_, of the neural network was calculated based on the input data of the input layer, *X*_*i*_, as in equation (8), where *f* is the activation function in the neural node. In this case study, the log-sigmoid function is used as an activation function. The errors between the output and expected values are utilized to update the weights and bias values in the backpropagation process. The output delta for hidden layers is calculated in equation (9). The weight vectors are updated iteratively in equation (10) with the learning rate value. The BPNN could be used to predict the leakage current of the insulator when the training process is completed with different independent datasets. However, the random initiative weight and bias values will affect the accuracy and convergent velocity, leading to a local minimum. Therefore, the BPNN could be used with other optimization algorithms to improve effectiveness and accuracy.

(8) $$\begin{array}{c} Y_{i}=f\left(\sum_{i=1}^{n}w_{ji}X_{i}+b_{ij}\right), \end{array}$$

(9) $$\begin{array}{c} \delta_{j}^{i}=f^{\prime }\left({\left(w_{i}^{j}\right)}^{T}X^{j-1}\right), \end{array}$$

(10) $$\begin{array}{c} w_{i}^{j+1}=w_{i}^{j}-\eta \delta^{j}X^{j-1}. \end{array}$$

### 2.5. Particle Swarm Optimization-Based Back Propagation Neural Network

**Figure 5:**
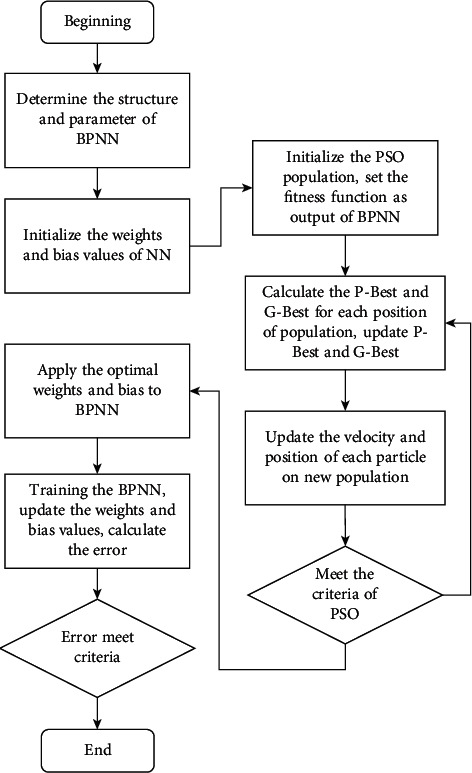
The flowchart of PSO combined BPNN.

The PSO-based neural network is deployed to forecast the leakage current in the online monitoring system. The PSO algorithm has fewer parameters, higher precision, and faster convergence. Therefore, the PSO algorithm is constructed to optimize the initial random values of weights and bias in the training process of neural networks. The proposed algorithm acquires complete advantage aspects of PSO and inherent the predicting capacity of BPNN. The diagram of the PSO algorithm combined with NN is clearly illustrated in Figure 5. In the first step, some essential parameters of PSO and the structure of NN are defined. The setting parameters of the PSO algorithm, the fitness function, and the population of PSO are initialized, and the initial random positions of each particle are generated. The personal best fitness and the global optimum fitness value, P-Best, and G-Best are calculated. The fitness function was updated based on the new particle's position in the next step. The local optimum of each individual in the population, the P-Best, and the G-Best are calculated and updated. The process is repeated in step 2 until the number of maximal iterations is reached, and the final value of G-Best is utilized as the initial weights and bias values for the neural network. The neural network uses the weights and bias values from the PSO process as initial values in the next step. The input parameters, the meteorological data, and the extracted surface spark data are normalized before applying them to the neural network. The predicting value is the normalized leakage current. In the backpropagation process, the neural network updated the weights and bias values during the training operations. The training operation finishes when the stopping criteria are reached. The final steps calculate the mean square error between the predicting and targeting values to assess the PSO-based neural network performance. The mean square error at the neural network's output is utilized as the fitness function of the PSO-based NN. The whole process of PSO-based neural networks is clarified clearly in Figure 5.

### 2.6. Hybrid of Particle Swarm Optimization- and Gene Algorithm-Based Neural Network

In recent years, the combination of GA, PSO, and neural networks, called a hybrid of particle swarm optimization- and gene algorithm-based neural networks, obtained much valuable research. The PSO and GA's applications solve lots of substantial combinational optimization problems. Although the hybrid PSO-GA inherits both advantages of the two algorithms, they require intensive computational resources and take a considerable amount of performing time [50]. However, the combination between PSO and GA could significantly decrease the number of iterations and increase the probability of achieving optimal convergence. In hybrid PSO-GA, these two algorithms work with the same population, initially randomly generated as individuals. These individuals are regarded as chromosomes in the GA algorithm and as particles in PSO. In a neural network, the number of hidden layers and the neural nodes of each layer are established in advance. In addition, the learning parameters in the PSO algorithm and the crossover and mutation probability in the GA algorithm are assigned in advance. After the initialization of the first generation, new individuals are created for the next generation by the crossover process and mutation operations. In each iteration, after the fitness functions of each individual in the population are calculated, the top best fitness individuals are selected as elites. The GA algorithm will reproduce the elite individuals directly to the next generation by crossover and mutation operation. These enhancement operations minimize the maturing phenomenon in nature and transfer these elite individuals to the next generation. The crossover and mutation processes use these enhanced individuals as parents to generate offspring, achieving better performance than original parents. The enhanced individuals from the GA algorithm continue to be handled by the PSO algorithm. In PSO, individuals enhance themselves based on their best personal cognition and global interactions within the population. By applying the PSO algorithm to elite individuals, premature convergence could be avoided to increase searchability. Each individual in the next generation is occupied by the elite chromosomes of the GA algorithm and enhanced individuals of PSO algorithms. The relevant operations are presented as follows:

  Step 1: determine the number of hidden layers and neurons for the neural network, and define the number of input and output. Define the training goal and the number of iterations. The mean square error (MSE) is utilized as a fitness function for PSO and GA.
  Step 2: initialize the weights and bias values for the neural network. Each vector of weight and bias values is an individual of the population. In this step, some setting parameters of PSO and GA are established: *n*Pop: The number of populations of PSO; MaxIt: the maximum number of iterations; VarMin and VarMax: the lower and upper boundary of position for each PSO population; VelMin and VelMax: the lower and upper boundary of velocity for each PSO population; *w* and *w*damp: the inertia weight and inertia weight damping ratio, *C*_1_ and *C*_2_: define the personal and global learning coefficient; *P*_*c*_ and *P*_*m*_: define the crossover and mutation percentage. After defining some important hybrid PSO-GA parameters, the PSO population is initialized.
  Step 3: calculate the fitness function for each individual, then update the *P*-Best for all individuals and *G*-Best values for the whole population.
  Step 4: apply the roulette wheel selection to the population based on their fitness values. Apply the crossover to the selected parents who generate new chromosomes for the next generation. Apply the mutation process to the selected population and calculate the fitness function for new mutative and crossover populations. Merge the new population with the previous population. Keep the optimal particle *n*Pop population with the best cost values of fitness functions.
  Step 5: update the velocity and position for each population. Compare the fitness values with the best personal fitness value of the particle. Update the *P*-Best and *G*-Best if the new fitness value is better than the old individual and global best-known value.
  Step 6: repeat step 3 until the process meets PSO criteria.
  Step 7: achieve the optimal weight and bias value for the neural network, apply the optimal solution, which is *G*-Best, to the backpropagation neural network.
  Step 8: train the neural network with the optimum solution, and update the weight and bias value due to the backpropagation of the neural network.
  Step 9: stop the training process when the error condition is satisfied or the maximum iterations are reached.

**Figure 6:**
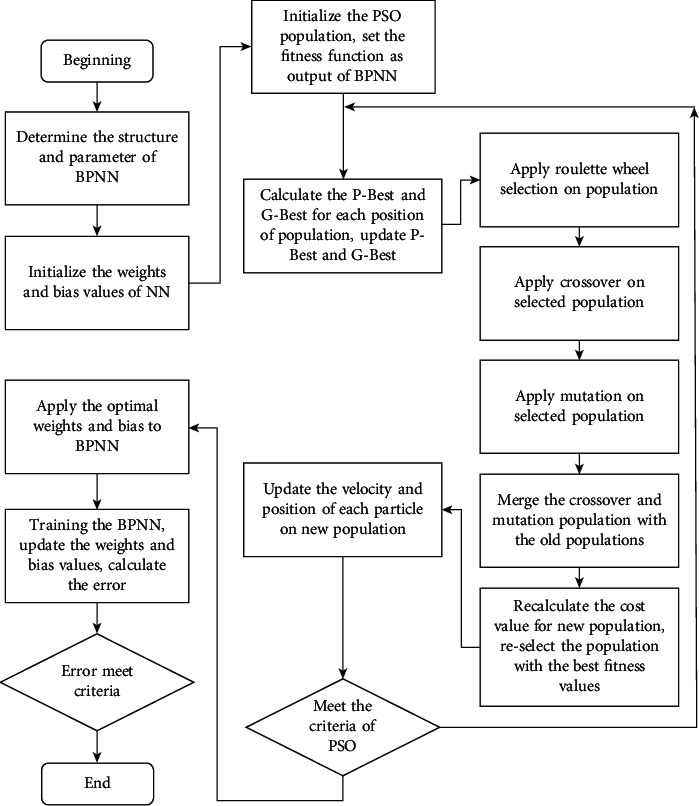
The hybrid PSO-GA-based neural network flowchart.

The final result will be the optimal solution for leakage current prediction. The PSO and GA have reasonably updated the hybrid algorithm's inertia weight and bias values and exchanged best-fit solutions. Therefore, this combination enhances the search space in local and global search capability, improves the convergent velocity, and increases the optimization capability in predicting leakage current. For clarity, the hybrid PSO-GA-based neural network flowchart operation is further illustrated in Figure 6.

### 2.7. Error Metric

The relative mean square error (MSE), the coefficient of determination, and the mean absolute percentage error (MAPE) are implemented to judge the effectiveness of predicting models. The MSE is a general factor to measure the differences between the predicting value, ${\hat{y}}^{p}$, and measuring values, *y*^*p*^, of leakage current. The MSE is calculated based on the difference between predicting and measuring values as equation (11), and the unit is *mA*^2^ for leakage current.

(11) $$\begin{array}{c} \mathrm{MSE}=\frac{1}{n}\sum_{1}^{n}{\left({\hat{y}}^{p}-y^{p}\right)}^{2}\left(mA^{2}\right). \end{array}$$

The coefficient of determination measures the proportion of predictable variance of dependent values, also known as *R* squared, as defined in the following equation.

(12) $$\begin{array}{c} R^{2}=1-\frac{{\sum_{i=1}^{N}\left(y_{i}^{p}-{\hat{y}}^{p}\right)}^{2}}{{\sum_{i=1}^{N}\left(y_{i}^{p}-\bar{y}\right)}^{2}}. \end{array}$$

The MAPE measures the percentage error between the predicting value, ${\hat{y}}^{p}$, and measuring values, *y*^*p*^, of leakage current. This error parameter evaluates the accuracy of the predicting model and is defined in equation (13). These error metrics are calculated and used to evaluate the performance and effectiveness of different models in predicting leakage current.

(13) $$\begin{array}{c} \mathrm{MAPE}=\frac{100}{n}\sum_{1}^{n}\left|\frac{{\hat{y}}^{p}-y^{p}}{y^{p}}\right|. \end{array}$$

## 3. Simulation Results

The proposed hybrid algorithm is utilized to illustrate the applicability in predicting leakage current. The meteorological data and surface spark data of 69 kV and 161 kV transmission towers are used as the input data models. All the training and validating input and output data are normalized before being utilized in the predicting model using max–min normalization.

The mean square error is utilized as a fitness function to evaluate the optimal searching solution of hybrid PSO-GA-based neural network, PSO-based neural network, and BPNN. In this case, the total iterations are 400, with the first 200 iterations for PSO and the last 200 iterations for BPNN. The setting population for both PSO and hybrid PSO-GA is 200 individuals. The personal and global learning coefficients are 2.0 and 2.5, respectively. The value of crossover percentage and mutation percentage in GA are 0.8 and 0.2, respectively. Because all these setting parameters have no specific criterion for regulation, these optimum values are utilized based on the trial-and-error procedures. The summary of optimal values for developed hybrid algorithms are presented in Table 3. All proposed algorithms use the MSE in the neural network the output as the fitness function. The simulation results of hybrid PSO-GA algorithms with the same structure and parameter compared with other persistent models in different data models are shown in Table 4.

The simulation results for data model 1 have shown that the BPNN model achieved 6.67*e* − 4, 9.55%, and 0.9189 for the MSE, MAPE, and *R*^2^, respectively, for 69 kV data. In the 161 kV data, the BPNN attained 10.7*e* − 4, 14.88%, and 0.8877 for the MSE, MAPE, and *R*^2^, respectively. Compared with the BPNN, the PSO-based NN algorithm acquired the 6.495*e* − 4, 9.62%, 0.9154 in the 69 kV data and 9.74*e* − 4, 13.65%, and 0.8982 in the 161 kV for the MSE, MAPE, and *R* square, respectively. In the hybrid PSO-GA-based NN, the simulation results show that MSE, MAPE, and *R* square values are 6.22*e* − 4, 9.5%, and 0.9190 for 69 kV data and 9.37*e* − 4, 13.48%, and 0.9020 for 161 kV data. Compared with BPNN and PSO-based NN, the PSO-GA-based NN achieves a maximum improvement of 6.75% and 12.43% for MSE, 1.25% and 9.41% for MAPE, and 0.39% and 1.61% for *R* square, in 69 kV and 161 kV, respectively. From the simulation results of data model 2, the BPNN model achieved 1.8207*e* − 4, 5.18%, and 0.9521 for the MSE, MAPE, and *R*^2^, respectively, for 69 kV data. For the 161 kV dataset 2, the BPNN acquired 1.5345*e* − 04, 5.23%, and 0.9732 for the MSE, MAPE, and *R*^2^, respectively. In the PSO-based NN model, the MSE, MAPE, and *R*^2^ for 69 kV are 1.51*e* − 4, 4.97%, and 0.9792, respectively. In the 161 kV data model 2, the PSO-based NN model achieved 1.25*e* − 4, 5%, and 0.9849 for MSE, MAPE, and *R*^2^. In addition, the PSO-GA-based neural network has achieved a maximum improvement of 38.54% and 30.28% for MSE, 10.62%, and 12.62% for MAPE, 3.41%, and 1.6% for *R* square in 69 kV and 161 kV data, respectively. Comparing the simulation results between data models, data model 2 always achieved better performance and higher accuracy, with a maximum improvement of 39.28% and 12.62% for MSE and MAPE, respectively. The simulation results between data models have proved that the surface spark data play an essential role in improving the ability to search the global solution during the training process. Besides, the surface spark data also strongly correlate with the leakage current. In addition, comparing the simulation results between data models, the combination between the surface spark data and the weather parameters has better effectiveness and higher stability in predicting the leakage current of insulators. The BPNN has achieved the minimum values of MSE and MAPE and the high value of *R*^2^, which are the local minimum in predicting the leakage current. The PSO-based NN has obtained a more substantial global search capability, improving the assignment solution for complex problems. However, the hybrid PSO-GA-based NN method has performed better for searching global optimum in predicting the leakage current of insulators comparing other models. The simulation results of hybrid PSO and GA get the better values of error metrics, which are the MSE, MAPE, and *R* square in both 69 kV and 161 kV data. With the surface spark data in 69 kV, the simulation results get the maximum improvement of MSE, MAPE, and *R* square as 38.54%, 10.62%, and 3.41%, respectively. In the 161 kV data, the hybrid PSO and GA outperform the PSO-based neural network and BPNN with better values of MSE, MAPE, and *R*^2^ with a maximum improvement of 39.28%, 12.62%, and 1.61%, respectively. Therefore, the hybrid PSO-GA method has a more substantial global optimal search capability than the standard PSO-based NN algorithm and BPNN. The hybrid PSO-GA could achieve a successful global search, which provides better accurate control for the neural network in training results. Therefore, the hybrid PSO-GA combined with enhanced surface spark data could predict the leakage current of insulators with higher accuracy and better effectiveness.

**Figure 7:**
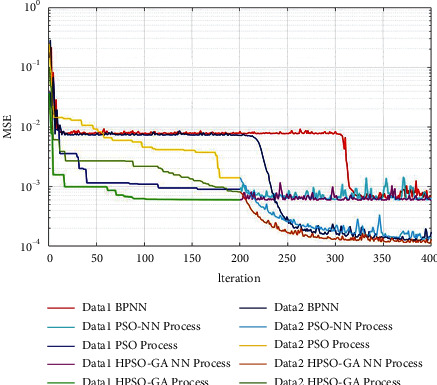
The MSE of training process of 69 kV data in different predicting algorithms and datasets.

**Figure 8:**
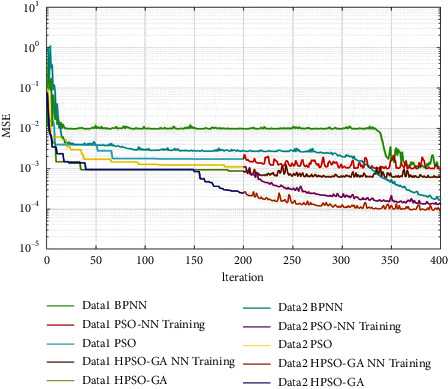
The MSE of training process of 161 kV data in different predicting algorithms and datasets.

The training processes were illustrated with the same setting parameters to show the effectiveness and efficiency of hybrid PSO-GA-based neural networks over PSO-based neural networks and BPNN. Figures 7 and 8 show the training process of BPNN, PSO-based NN, and hybrid PSO-GA-based neural network in 400 iterations between data models using MATLAB. In three algorithms, the MSE is utilized as the fitness function. In the BPNN training process, the maximum iteration is set as 400, the same as other models. In the PSO-based NN training process, the PSO algorithm is applied for the first 200 iterations to optimize the neural network's initial weights and bias values. In the following 200 iterations, the backpropagation neural network is applied to update the weights and bias values during the training process. Similarly, in the hybrid PSO-GA-based NN, the PSO-GA is utilized in the first 200 iterations; and the subsequent 200 iterations are used for the training process of the backpropagation neural network. Figures 7 and 8 have clearly illustrated the faster convergence in searching global solutions of the proposed algorithm compared with BPNN and PSO-based neural networks. In the first 200 iterations, the PSO and hybrid PSO-GA also get better convergent velocity to global optimization than BPNN. In the subsequent 200 iterations, the BPNN process of PSO-GA can search the global optimization, which achieved better MSE values than other models. Therefore, the proposed hybrid PSO-GA algorithm is designed by iteratively updating the weight and bias values; and the process of exchanging best fitness solutions between PSO and GA has optimized the global search capability in predicting the leakage current of insulators. The combination of GA and PSO has improved global and local search capability. This modification algorithm could obtain better optimization solutions in the neural network training process's randomly generated initial weight and bias values. Compared with PSO-based NN and BPNN, the hybrid PSO-GA-based NN could improve the success rate of finding the optimal solution and achieve better accuracy and effectiveness in predicting leakage current.

To prove the effectiveness and efficiency in predicting the leakage current of hybrid PSO-GA compared with the PSO-based neural network and BPNN, Table 5 shows the absolute relative error between the predicting and measured leakage current of insulators in three models. For data model 1, the BPNN attained 6.23% and 6.07% relative error for 69 kV and 161 kV data. The PSO-based NN performs better with 5.95% and 5.36% than BPNN. However, the hybrid PSO-GA-based NN could achieve the best relative errors, 5.62% and 4.89% for 69 kV and 161 kV data model 1. In data model 2, the BPNN predicting results show 5.15% and 4.65% relative error between predicting and measuring values for the 69 kV and 161 kV datasets, respectively. However, the PSO-based neural network performs better with 4.23% and 3.92% relative error for the 69 kV and 161 kV datasets 2. Compared with other models, the PSO-GA-based neural network has the best values of relative error, which are 4.01% for the 69 kV dataset and 3.75% for the 161 kV dataset 2. This hybrid PSO-GA-based NN has better optimal fitness values and demonstrates its capability to resolve the leakage current problem. This combination of the two algorithms reasonably updates the inertia weight and bias values. Besides, combining surface spark data could exchange best-fit solutions between PSO and GA to optimize the neural networks' training process. The exchange best-fit solution also strengthens the hybrid algorithm's global optimum and local search capability in predicting leakage current.

The experiment results demonstrate the effectiveness of the combined PSO-GA-based BPNN for predicting the leakage current of insulators with different evaluating benchmarks. This research provides the following contributions in predicting the leakage current of 69 kV and 161 kV insulators:

1. The combined PSO-GA-based NN outperforms the traditional PSO-based NN in both traditional and enhancement data. Compared with the persistent PSO-based NN model, in the traditional data, which contains only meteorological information, the developed algorithm achieves maximum enhancements of 4.23% MSE, 1.25% MAPE, 0.39% R-square for 69 kV data, and 3.8% MSE, 1.25% MAPE, 0.42% *R*-square for 161 kV data. For the novel data, which include the weather factors and enhanced inputs, the hybrid algorithm obtains more significant improvements of 26.19% MSE, 6.84% MAPE, 0.55% *R*-square for 69 kV data, and 25.75% MSE, 8.6% MAPE, 0.4% *R*-square in the 161 kV data than the PSO based NN method. The hybrid PSO-GA methodology is more accurate in predicting the leakage current than the PSO algorithm.
2. The developed PSO-GA-based NN outperforms the traditional BPNN with considerable accuracy and performance in both collected data models. Compared with BPNN, the hybrid algorithm achieves the most remarkable improvements of 38.54% MSE, 10.62% MAPE, 3.41% *R*-square in the 69 kV data and 39.28% MSE, 12.62% MAPE, 1.61% *R*-square for 161 kV data, which proves the effectiveness in adding the PSO-GA in the neural network process.
3. The enhanced data outperform the traditional data, which achieves higher accuracy and better stability in predicting the leakage current of high voltage insulators. The additional enhanced data prove higher effectiveness and better accuracy, providing a better the correlation with the leakage current of 69 kV and 161 kV insulators.
4. The predicting leakage currents of the 161 kV insulator are more accurate than the 69 kV insulator because of the higher level of leakage currents. Therefore, the developed algorithm could be utilized in predicting the high voltage of insulators, not only in this project but also in Taiwan.

Although the PSO-GA-based NN outperforms the traditional algorithms in predicting the leakage current, the developed algorithm also needs deeper evaluation and analysis with different data models. In future research, the authors will investigate the developed algorithm in other electrical projects to analyze the performance better.

## 4. Conclusions

The combination between PSO and GA provides some critically important steps for exchanging best-fit solutions in neural network optimization and makes this hybrid algorithm more efficient than BPNN and PSO-based neural networks. Primarily when the surface spark data were utilized with traditional input parameters to deal with the optimization in predicting leakage current of insulators. This combinative algorithm could be applied effectively in online monitoring systems, enhancing system efficiency, reducing operational maintenance costs, and easing the computational burden. This paper has successfully established a hybrid PSO-GA-based neural network and surface spark data to optimize the accuracy of the predicting system to utilize in the online monitoring system. The exchange of best fitness between PSO and GA algorithm has been proved to improve both global and local search capability. The results in the 69 kV and 161 kV transmission data simulation demonstrate the superiority of hybrid PSO-GA-based neural networks over BPNN and PSO-based neural networks with the support of surface spark data as enhancement input parameters. The experiments proved that the hybrid PSO-GA achieved the maximum improvements of 39.28%, 12.62%, and 3.41% for MSE, MAPE, and *R* square. Therefore, the developed methodology could predict the leakage current on the online monitoring system with better accuracy and higher stability [51–54].

## Figures and Tables

**Table 1:** The summary of weather parameter.

Values	Temperature (°C)	Humidity (%)	Dew point (°C)	Wind speed (m/s)	Win direction (Rad)	Air pressure (hPa)	Rainfall (mm/h)
Maximum	33.52	98.00	28.60	13.20	0.00	1024.02	11.80
Minimum	9.28	41.00	0.75	0.00	337.50	1001.47	0.00
Average	21.71	85.25	19.06	5.46	110.63	1014.73	0.00
Standard deviation	4.65	7.37	4.41	2.59	99.35	4.54	0.33

**Table 2:** The Pearson correlation between input parameters and the leakage currents.

	Leakage current	Percent SSDP	Brightness SSDP	Temperature	Humidity	Dew point	Wind direction	Wind speed	Air pressure	Rainfall
Leakage current	**1.00**	**0.22**	**0.67**	**−0.15**	**0.22**	**−0.44**	**−0.17**	**0.14**	**0.13**	**0.31**
Percent SSDP	**0.22**	**1.00**	**0.35**	**0.23**	0.08	**0.32**	0.00	−0.07	−0.06	−0.06
Brightness SSDP	**0.67**	**0.35**	**1.00**	−0.06	**0.50**	**0.25**	−0.06	−0.05	0.07	0.08
Temp	**−0.15**	**0.23**	−0.06	**1.00**	**−0.50**	**0.83**	**0.21**	0.04	**−0.13**	**−0.20**
Hum	**0.22**	0.08	**0.50**	**−0.50**	**1.00**	0.07	**−0.11**	−0.09	**0.19**	**0.10**
Dew point	**−0.44**	**0.32**	**0.25**	**0.83**	0.07	**1.00**	**0.17**	−0.01	−0.03	**−0.16**
Wind direction	**−0.17**	0.00	−0.06	**0.21**	**−0.11**	**0.17**	**1.00**	**0.40**	0.02	−0.03
Wind speed	**0.14**	−0.07	−0.05	0.04	−0.09	−0.01	**0.40**	**1.00**	**−0.11**	−0.02
Air pressure	**0.13**	−0.06	0.07	**−0.13**	**0.19**	−0.03	0.02	**−0.11**	**1.00**	−0.01
Rainfall	**0.31**	−0.06	0.08	**−0.20**	**0.10**	**−0.16**	−0.03	−0.02	−0.01	**1.00**

**Table 3:** The optimum values for the hybrid PSO-GA algorithms.

Optimum values for hybrid model		Value
The PSO parameters	Boundary of variables	[−5; 5]
	Max iterations	200
	Number population	200
	Boundary of velocity	[−1; 1]
	Inertia weight damping ratio	0.99
	Personal learning coefficient	2.0
	Global learning coefficient	2.5

The GA algorithm	Crossover percentage	0.8
	Number of offspring	160
	Extra range factor for crossover	0.4
	Mutation percentage	0.2
	Number of mutants	40
	Mutation rate	0.1

**Table 4:** The comparison of simulation results between models.

							Percent improvement (%)					
	MSE		MAPE (%)		*R* square		MSE		MAPE (%)		*R* square	
	161 kV insulators						69 kV insulators					
	Data 1	Data 2	Data 1	Data 2	Data 1	Data 2	Data 1	Data 2	Data 1	Data 2	Data 1	Data 2
BPNN	6.67*E* − 04	1.82*E* − 04	9.55	5.18	0.9189	0.9521	6.75	38.54	0.52	10.62	0.01	**3.41**
PSO-based NN	6.50*E* − 04	1.52*E* − 04	9.62	4.97	0.9154	0.9792	4.23	26.19	1.25	6.84	0.39	0.55
PSO-GA NN	**6.22*E* − 04**	**1.12*E* − 04**	**9.5**	**4.63**	**0.919**	**0.9846**						

	161 kV insulators						161 kV insulators					
	Data 1	Data 2	Data 1	Data 2	Data 1	Data 2	Data 1	Data 2	Data 1	Data 2	Data 1	Data 2
BPNN	1.07*E* − 03	1.53*E* − 04	14.88	5.23	0.8877	0.9732	12.43	**39.28**	9.41	**12.62**	1.61	1.60
PSO based NN	9.74*E* − 04	1.25*E* − 04	13.65	5	0.8982	0.9849	3.80	25.75	1.25	8.60	0.42	0.40
PSO-GA NN	**9.37*E* − 04**	**9.32*E* − 05**	**13.48**	**4.57**	**0.902**	**0.9888**						

**Table 5:** The absolute relative error between predicted and measured values of leakage current.

	Absolute relative error (%)			
	69 kV		161 kV	
	Data 1	Data 2	Data 1	Data 2
BPNN	6.23	5.15	6.07	4.65
PSO based NN	5.95	4.23	5.36	3.92
PSO-GA based NN	**5.62**	**4.01**	**4.89**	**3.75**

## Data Availability

The research data used to support the findings of this study were supplied by ETRC (Electric Technology Research Center) under license and so cannot be made freely available. Requests for access to these data should be made to the corresponding author.
